# Supplementary material for: Ginsenoside Compound K Protects against Obesity through Pharmacological Targeting of Glucocorticoid Receptor to Activate Lipophagy and Lipid Metabolism
Source: Pharmaceutics. 2022 Jun 2;14(6):1192. doi: 10.3390/pharmaceutics14061192 (PMC9231161; doi:10.3390/pharmaceutics14061192)
Supplement: Supplementary file 1 [file pharmaceutics-14-01192-s001.zip › pharmaceutics-1686927-supplementary.pdf]

pharmaceutics

MDPI

Supplementary Materials: Ginsenoside Compound K Protects Against Obesity through Pharmacological Targeting of Glucocorticoid Receptor to Activate Lipophagy and Lipid Metabolism

Siwen Yang [1], Ting Liu [1], Chenxing Hu [1], Weili Li [1], Yuhan Meng [1], Haiyang Li [1], Chengcheng Song [1], Congcong He [2], Yifa Zhou [1,*], Yuying Fan [1,*]

CK reduces body weight and blood glucose in obese mice

The leptin-deficient obese mice (ob/ob) were subjected to different concentration of CK (5, 10, 20 mg/kg/d) for 5 weeks. The body weight and fasting blood glucose significantly decreased after 20 mg/kg/d CK treatment for 5 weeks (Figure S1a, b). In addition, the glucose tolerance test (GTT) and insulin tolerance test (ITT) were significantly ameliorated in a dose-dependent manner (Figure S1c, d).

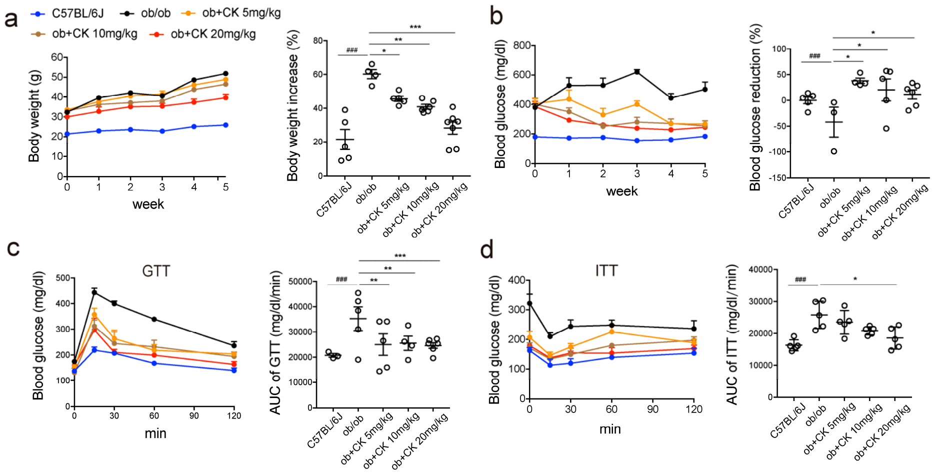

Figure S1. Metabolic profiles of the obese mice with different doses of CK treatment (n=4-8). (a) Body weight and its increase rate. (b) Fasting blood glucose and its decrease rate. (c) Glucose tolerance test and AUC. (d) Insulin tolerance test and AUC. Results represent mean ± SD. ###, $p < 0.001$; *, $p < 0.05$; **, $p < 0.01$; ***, $p < 0.001$.

CK exerts hypolipidemic activity in obese mice

The 20 mg/kg/d CK treatment did not affect food intake regardless of whether in the day or in the dark (Figure S2a). However, It reduced the weights of white adipose tissue and liver (Figure S2b). Moreover, CK treatment reduced respiratory exchange rate (RER), indicating that CK promoted fat consumption, whereas it did not promote carbohydrate consumption (Figure S2c). It also reduced the TG and NEFA levels in the liver (Figure S2d, e) and increased the phosphorylation levels of the insulin receptor and its substrate, indicating that CK increased insulin sensitivity in the liver (Figure S2f).

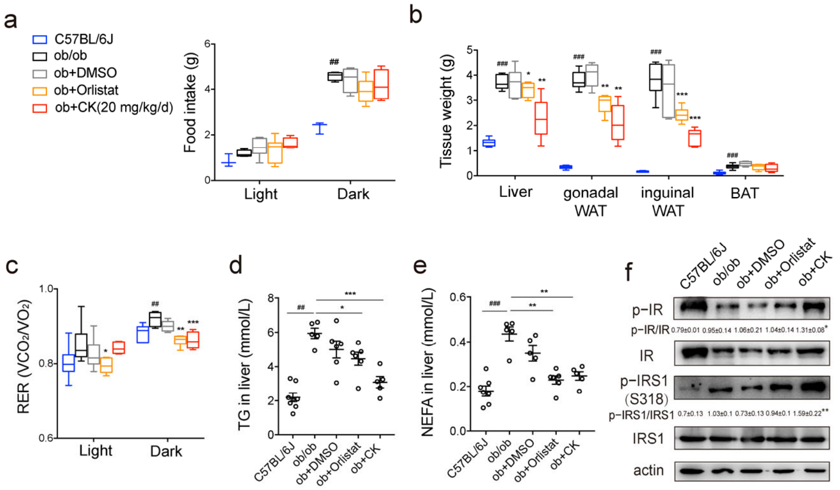

Figure S2. Metabolic profiles and lipid levels of obese mice after CK treatment. (a) Food intake. (b) Weight of indicated organs. (c) Respiratory entropy. (d) Triglyceride levels in liver. (e) Non-esterified fatty acid levels in liver. (f) Insulin signaling pathways were analyzed using western blotting of liver tissues. Statistics compare each value in compounds-treated groups to the one in ob/ob groups. Results are presented as mean ± SD. ##, $p < 0.01$; ###, $p < 0.001$; *, $p < 0.05$; **, $p < 0.01$; ***, $p < 0.001$.

The autophagy induction ability of CK

GFP-LC3 transfected HeLa cells were used to test the autophagy induction ability of CK. The number of autophagosomes with CK treatment was increased in dose-dependent manner. The EC50 values in cells treated with CK was 35 μM (Figure S3a). Moreover, ginsenoside CK exhibited the highest potential for activating autophagy not only in HeLa cells, but also in HepG2 and A549 cells. After the addition of the autophagy inhibitors bafilomycin A1 (BafA1, which blocks autophagosome and lysosome fusion), the degradation of p62 and conversion of LC3-I to LC3-II were significantly suppressed compared to that in the control (Figure S3b, c). The results further indicate that ginsenoside CK activates autophagy.

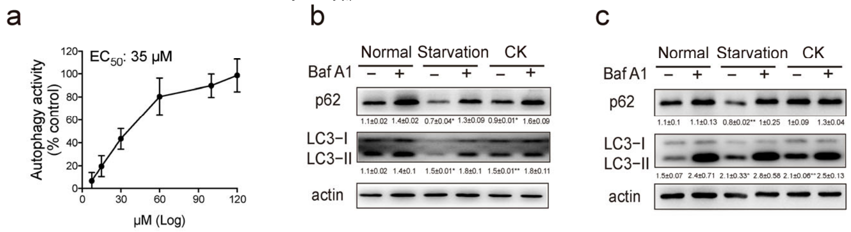

Figure S3. Ginsenoside CK induces autophagy. (a) EC_{50} of CK in autophagy induction. Quantification of GFP-LC3 puncta in GFP-LC3/HeLa cells after 3 h treatment of CK at indicated concentrations (n=100). Western blot detection of p62 and LC3 in HepG2 cells (b) or A549 cells (c) cultured in normal or starvation medium, or treated with CK in normal medium, in the presence or absence of BafA1 for 3 h (n=3). Results are presented as mean ± SD. *, $p < 0.05$; **, $p < 0.01$.
